# Abusive Supervision, Affective Commitment, Customer Orientation, and Proactive Customer Service Performance: Evidence From Hotel Employees in China

**DOI:** 10.3389/fpsyg.2021.648090

**Published:** 2021-04-15

**Authors:** Dexia Zang, Chang Liu, Yan Jiao

**Affiliations:** ^1^Department of Management and Human Resources, Business School, Hohai University, Nanjing, China; ^2^Department of Tourism Enterprises, College of Tourism and Service Management, Nankai University, Tianjin, China

**Keywords:** abusive supervision, affective commitment, customer orientation, proactive customer service performance, affective events theory

## Abstract

Abusive supervision is quite common in the service industry. Employees’ proactive customer service performance is essential for the long-term development of service enterprises. This study enriches the antecedents of proactive customer service performance from a new theoretical perspective by incorporating the analysis of abusive supervision into the theoretical framework and fills the research gap between customer orientation and proactive customer service performance. Based on Affective Events Theory and Social Cognitive Theory, this study established the structure equation model between abusive supervision and proactive customer service performance mediated by affective commitment and customer orientation. Utilizing structural equation modeling, a negative association between abusive supervision and proactive customer service performance was found, and affective commitment and customer orientation act as the mediators between abusive supervision and proactive customer service performance. In addition, the implications for future study were also discussed.

## Introduction

In the era of the service economy, consumers pay more attention to the service value and experience quality rather than the cheap price (Zhou Z., 2016). Service features like perishability and the simultaneity of production and consumption determine how the degree of direct involvement of employees in the entire process of service production, delivery, and consumption (Fitzsimmons and Fitzsimmons, 2001; Fynes and Lally, 2008), which gives employees’ attitudes and behaviors the potential to influence consumers’ perceived service value and their satisfaction (Kattara et al., 2008; Pollack, 2008). Therefore, the question of how employee attitudes should be guided and how their behaviors toward customers should be managed has become a hot topic for service enterprises and related researchers (Raub and Liao, 2012; Ye et al., 2019). The employees’ behaviors and proactive performances, such as predicting the demands of customers prospectively, improving service process initiatively, voicing ideas to other colleagues actively, and delivering “extra” service persistently, will increase the customers’ perceived service quality and satisfaction (Bitner et al., 1990).

In recent years, many studies have focused on employees’ proactive customer service performance (PCSP) (Ye et al., 2019; Cheng B. et al., 2020; Cheng T. et al., 2020), which is an individuals’ self-starting, long-term-oriented, and persistent service behavior beyond the explicitly prescribed performance requirements (Rank et al., 2007). There are some influencing factors for PCSP. It is significantly and positively associated with trait personal initiative, affective organizational commitment, task complexity, and participative leadership (Rank et al., 2007). It is also positively associated with self-efficacy at the individual level and initiative climate at the establishment level (Raub and Liao, 2012; Lau et al., 2017) and negatively affected by leader-member exchange differentiation (Cheng T. et al., 2020) and workplace mistreatment, such as workplace sexual harassment (Li et al., 2016). As far as the leadership style is concerned, some researchers have just explored it from the perspective of positive leadership, including participative leadership and transformational leadership (Jiang et al., 2020; Wu et al., 2020). In addition to positive leadership, in the service field, destructive leadership is also a common leadership style; in fact, destructive leadership is more likely to have a strong and lasting impact on employees’ behavior than positive leadership (Baumeister et al., 2001). But there are few studies that explore how destructive leadership influences PCSP (Lyu et al., 2016). Of many kinds of destructive leadership, abusive supervision is quite common, especially in the service industry (Lyu et al., 2016; Al-Hawari Mohd et al., 2020). Therefore, our study goes beyond the existing influencing factors of PCSP and focuses on abusive supervision to try to explore the relationship mechanism between abusive supervision and PCSP.

As we all know, abusive supervision is a kind of common cold violence in the workplace, especially in a Chinese context. There are some small-scale surveys showing that more than half of Chinese employees have been subject to abusive supervision (Liang et al., 2016; Shen et al., 2020). The largest scale survey on workplace cold violence in China is an online questionnaire survey conducted by *zhaopin*, one of China’s biggest human resource service agencies. Over 70% of the more than 10,000 participants encountered cold violence in the workplace, including abusive supervision, exclusion from certain opportunities, unreasonable assignments, and so on (Shen et al., 2020; Wang et al., 2020). Abusive supervision is defined as subordinates’ perceptions of the extent to which supervisors engage in the sustained display of hostile verbal and non-verbal behaviors, excluding physical contact (Tepper, 2000). It is fairly common in practice, which transfers a kind of negative influence and does harm to employees’ psychology, thereby affecting their work performance (Moin et al., 2020).

Previous studies mainly adopted the perspective of Conservation Of Resources Theory and Social Exchange Theory to explain the effect of leadership style on PCSP (Tuan, 2018; Ye et al., 2019). However, few studies have explored this relationship from the perspective of Affective Events Theory and Social Cognitive Theory. Affective Events Theory argues that work events affect employees’ work attitudes and then work behaviors in a “judgment-driven” manner (Weiss and Cropanzano, 1996). Negative events in the workplace are important emotional events that affect employees’ affective states and work attitudes (Bono et al., 2013). As a negative work event, abusive supervision from supervisors reduces employees’ emotional identities and weakens their affective commitments (Tillman et al., 2018). Affective commitment, as a kind of employees’ work attitude, affects their work behavior, such as customer orientation and PCSP, through the judgment drive. In addition, Social Cognitive Theory argues that individual behavior is influenced by cognition (Bandura, 1986) and self-regulation of their cognition and behavior by comparing their expectations and actual results (Bandura, 1991). Employees with low customer orientation think that their work is meaningless (Zhu et al., 2017) and have low psychological empowerment (Zeglat et al., 2014), which results in negative expectations for service provision (Shamsifar et al., 2019). They will consequently self-regulate their service behavior and reduce PCSP to meet psychological expectations (Schunk and DiBenedetto, 2020). Therefore, we try to explore the internal mechanism of abusive supervision on PCSP and the mediating role of affective commitment and customer orientation from the perspective of Affective Events Theory and Social Cognitive Theory.

This study investigates the influence of abusive supervision on PCSP empirically confirms the influencing mechanism of abusive supervision on PCSP further by establishing the structure equation model among related constructs. This study enriches research related to abuse supervision and PCSP based on Affective Events Theory and Social Cognitive Theory; it not only reveals the impact of destructive leadership on employees’ behaviors but also reveals the deep motivation of employees behaviors through the influencing mechanism of abusive supervision on PCSP, which can help to improve employees’ performance in the practice of service enterprises. Specifically, this study makes three contributions to the current service science knowledge about PCSP. First, this study enriches the antecedents of PCSP by bringing abusive supervision analysis into the theoretical framework. Because there are not many studies on the relationship between destructive leadership behavior and employees’ PCSP, this study expands the theory of abusive supervision by thoroughly analyzing its influence on PCSP in the service industry. Second, this study provides a new perspective from the Affective Events Theory by introducing affective commitment as an intermediary to enhance our understanding of the influence mechanism of abusive supervision on PCSP. Since the influence of leadership style on PCSP is rarely analyzed from the perspective of Affective Events Theory previously, this study provides a new theoretical perspective for the interpretation of PCSP antecedents. Based on this perspective, this study provides a theoretical interpretation of the mediating role of affective commitment in the relationship between abusive supervision and PCSP. Third, this study integrates Affective Events Theory and Social Cognitive Theory to explore the mediating role of customer orientation in the relationship between affective commitment and PCSP. Since there are few studies on the promoting effect of customer orientation on PCSP, this study provides a theoretical deduction for the influence of customer orientation on PCSP from the perspective of Social Cognitive Theory and explores their relationship empirically, which will fill the gap in this issue.

## Theoretical Framework and Hypotheses Development

### Theoretical Framework

The theoretical framework of this study is developed on the basis of Affective Event Theory. Focusing on the structure, causes, and consequences of work affective experiences, Affective Events Theory is considered to be an important theoretical basis for the study of affect, attitude, and behavior in an organizational context (Weiss Howard and Beal Daniel, 2005; Ghasemy et al., 2021). From the perspective of Affective Events Theory, some work environment features can lead to the occurrence of positive or negative work events, which will trigger employees’ positive or negative affective reactions and directly influence their work behaviors through the “affect-drive” or influence their attitude and then work behaviors through “judgment-drive” (Weiss and Cropanzano, 1996). Disposition moderates the relationship between affective work events and affective reactions (Weiss Howard and Beal Daniel, 2005; Ghasemy et al., 2021).

**FIGURE 1:**
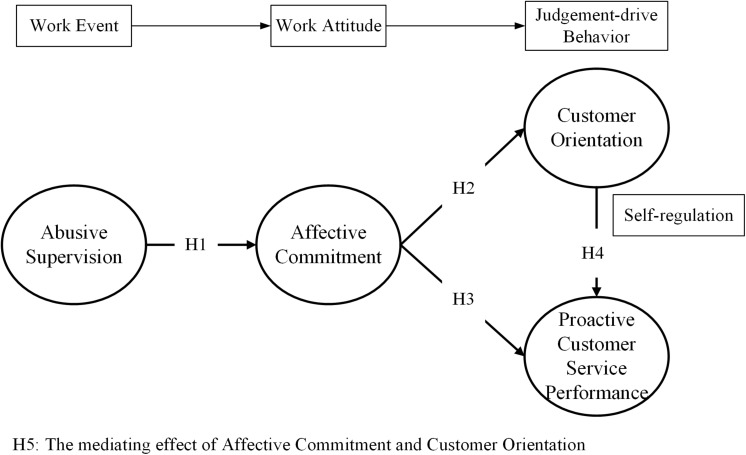
Theoretical framework and proposed research model.

Based on effective events theory (Ghasemy et al., 2021), this study establishes the theoretical framework shown in Figure 1, which explains the chain relationship between the work event, work attitudes, and judgment-driven behavior. Specifically, abusive management is a negative work event, which causes the employees’ negative work attitudes (Tillman et al., 2018), such as the reduction of affective commitment to the organization. Negative work attitude drives employees to make negative work behaviors through judgment (Bono et al., 2013; Ghasemy et al., 2021), such as reducing customer orientation and PCSP. In addition, as previous studies have proved that customer orientation positively affects organizational performance (Goad and Jaramillo, 2014; Li et al., 2020), this study further examines the impact of customer orientation on PCSP in the model. This is consistent with the view of self-regulation in Social Cognitive Theory, which advocates that individuals self-regulate their cognition and behavior according to the difference between the expected result and the actual result (Bandura, 1991; Schunk and DiBenedetto, 2020). Customer-oriented employees have good expectations of customer service performance (Shamsifar et al., 2019) and improve PCSP through self-regulation to achieve better service performance (Schunk and DiBenedetto, 2020).

### Hypotheses Development

Based on the theoretical framework, as shown in Figure 1, this study develops the following hypotheses through literature review to holistically explore the relationship among abusive supervision, affective commitment, customer orientation, and PCSP.

#### Abusive Supervision and Affective Commitment

According to Tepper’s definition, abusive supervision originates from a supervisor’s hostility of the superior to the subordinate. It is a subjective assessment of subordinates to their supervisors’ hostile verbal and non-verbal behaviors (i.e., behaviors that are not physical contact) (Tepper, 2000). Since this is the subjective perception of subordinates, different subordinates may have different perceptions of the same abusive behavior (Tepper, 2007). As a component of commitment, affective commitment is a kind of employees’ attachment to the organization, which expresses the individuals’ emotional connection, identification, and involvement of the individual with the organization (Allen and Meyer, 1990). According to Affective Events Theory, work events affect employees’ work attitudes by influencing their affective reactions. Abusive supervision, as a work event perceived by employees, can affect employees’ affective reactions, and then work attitudes such as affective commitment. More specifically, there is a social exchange relationship between employees and leaders based on reciprocity (Tepper et al., 2008; Jung and Takeuchi, 2019). However, when abusive supervision occurs, this leader–member exchange relationship would be destroyed (Choi et al., 2019; Park and Kim, 2019). Abused employees cannot get the internal rewards they expect, resulting in a perception of breaking a psychological contract (Park and Kim, 2019), which can lead to their distrust of supervisors, psychological frustration (Zhou L., 2016; Chen and Wang, 2017), and, eventually, a negative affective state whilst at the organization (Mackey et al., 2018; Greco et al., 2019). These negative affective state would then reduce employees’ emotional identity (Moin et al., 2020; He et al., 2021) and weaken their affective commitment (Xu et al., 2012; Wang and Peng, 2015). Besides, distributive justice has a positive impact on affective commitment (Aguiar-Quintana et al., 2020), and abusive supervision can lead to a decrease in employees’ affective commitment by affecting distributive justice. Abusive supervision would lead to distributive injustice (Tepper, 2007), which would make employees frustrated and angry, thus reducing their affective commitment (Tepper, 2000).

Although some studies have found that abusive supervision has a negative impact on affective commitment (Tepper et al., 2008; Yu et al., 2016), few studies have discussed the relationship between abusive supervision and affective commitment from the perspective of distributive justice. Moreover, the studies on the impact of abusive supervision on affective commitment rarely involve the context of the hotel industry. Therefore, this study assumes that in the service industry, including the hotel industry, abusive supervision may be also negatively related to affective commitment, as interpreted as Hypothesis 1.

H1: There is a negative association between Abusive Supervision and Affective Commitment.

#### Affective Commitment and Customer Orientation

Customer orientation was first defined as satisfying customer needs at the level of the salesperson–customer interaction to seek and build long-term customer relationships (Saxe and Weitz, 1982). Since then, researchers have conceptualized customer orientation either from the organizational or individual perspective. Customer orientation in this study is the a defined from the individual perspective, that is, it refers to individual employees striving to satisfy customers’ needs and desires through quality service (Donavan et al., 2004).

According to the Affective Events Theory, employees’ affective commitment in service-oriented enterprises affects their customer orientation. Previous studies have found that an employee gains a positive sense of belonging and self-worth through affective commitment (He et al., 2011), thereby enhancing their sense of identity with organizational goals and values and their organizational loyalty (Joshi and Randall, 2001), which can effectively motivate employees to engage in behaviors that are beneficial to achieving organizational goals, such as customer-oriented behaviors in order to improve customer satisfaction (Noor et al., 2012; Lombardi et al., 2019). On the other hand, studies have found that the higher employees’ affective commitment, the higher job satisfaction they have (Joshi and Randall, 2001; Ribeiro et al., 2020), and employees with higher levels of job satisfaction are more committed to customer service, more inclined to enjoy the process of serving customers and meet customer needs more fully (Donavan et al., 2004), and more likely to be customer-oriented (Ahmad and Lim, 2015). In short, affective commitment has a positive effect on employees’ customer orientation (Ribeiro et al., 2020). Therefore, we can propose the following hypothesis.

H2: There is a positive association between Affective Commitment and Customer Orientation.

#### Affective Commitment and Proactive Customer Service Performance

Proactive customer service performance includes proactive idea implementation and proactive problem solving (Parker et al., 2006). It is characterized as a self-starting, long-term oriented, and forward-thinking approach to service delivery, which is beyond the explicitly prescribed performance requirements (Rank et al., 2007).

According to Affective Events Theory, employees’ affective commitment has an impact on their PCSP. Researchers have found that employees’ affective commitment has a positive impact on their extra-role and task performance (Loi et al., 2012). Employees with strong affective commitments are more likely to make discretionary behaviors, which are not specified within the scope of their job responsibilities but would contribute to the organization (Paulin et al., 2006), such as improving customer satisfaction, maintaining customer relationships, and improving organizational reputation (Paulin et al., 2006; Dhar, 2015) by improving services (Zeithaml et al., 1990). Therefore, affective commitment is positively related to customer service quality (Dhar, 2015; Jaiswal and Dhar, 2016) and proactive service performance (Rank et al., 2007). Based on the current research, we can propose the following hypothesis:

H3: There is a positive association between Affective Commitment and PCSP.

#### Customer Orientation and Proactive Customer Service Performance

According to the self-regulation view of Social Cognitive Theory, employees’ customer orientation has a positive impact on their PCSP. Studies have shown that customer orientation positively affects organizational performance (Goad and Jaramillo, 2014; Li et al., 2020). However, there are few studies on the relationship between customer orientation and PCSP. According to previous studies, customer orientation was positively related to employees’ psychological empowerment (Zeglat et al., 2014), would improve employees’ job satisfaction and job involvement (Gazzoli et al., 2012), prompt employees to actively understand the actual needs of customers (Hamzah et al., 2016), and then improve work performance by developing service capabilities (Tang, 2014) and promoting service innovation (Cheng and Krumwiede, 2012). Some researchers considered that employees with higher customer orientation will have a higher level of role breadth self-efficacy (Huo et al., 2019). They would be more willing to think their work meaningful, believe in their own impact, and feel more self-confident to fulfill their work, and they would thus become more competent when it comes to satisfying all kinds of customers’ needs. This will therefore result in higher PCSP (Zhu et al., 2017). For these reasons, the following hypothesis is presented.

H4: There is a positive association between Customer Orientation and PCSP.

#### The Mediating Effect of Affective Commitment and Customer Orientation

From the perspective of Affective Events Theory, some work environment features can lead to the occurrence of positive or negative work events, which will trigger employees’ positive or negative affective reactions, and affect their attitude and then work behaviors through “judgment-drive” (Weiss and Cropanzano, 1996). As a negative work event, abusive supervision of superiors would trigger the negative affective reactions of employees, which may cause employees to reduce their affective commitment to the organization, thereby losing their willingness to serve customer-oriented, and ultimately leading to a reduction in their PCSP level. Therefore, the following hypothesis can be proposed.

H5: Affective Commitment and Customer Orientation sequentially mediate the relationship between Abusive Supervision and PCSP.

## Materials and Methods

### Sample and Procedure

Based on the thorough literature review, a quantitative questionnaire was developed with the aim of addressing the postulated hypotheses, and it was translated into Chinese by a linguist with expertise in English and Chinese in order to ensure terminological accuracy. The questionnaire consisted of three sections. The first section was the employees’ scale, which included the tools measuring abusive supervision, affective commitment, and customer orientation, where employees were asked to evaluate the actual levels of the above issues under investigation with the utilization of a 5-point Likert scale ranging from 1 strongly disagree to 5 strongly agree. The second section was the supervisors’ scale, which measured proactive customer service performance by adopting Rank’s 7-item scale, where the corresponding supervisors evaluated their subordinates’ proactive performance by using a 5-point Likert scale (1 = strongly disagree to 5 = strongly agree). The third section included some demographic questions where the subordinates were required to answer.

This survey was conducted in 16 hotels and restaurants located in Shandong Province from May to August of 2015. Since the target respondents of the survey are the front-line service staff in hotels and restaurants, the investigation method of matched pairs of employees and their immediate supervisors was adopted. Based on the list of supervisors and subordinates provided by the hotel, the paper questionnaires of employees and supervisors are labeled with paired codes and are put in a cover letter in advance. The header of the questionnaire states that it is an anonymous questionnaire to ensure that the respondent responds to the questionnaire without psychological pressure. The survey is divided into two parts and conducted at the same time. The subordinates rate their supervisors’ abusive supervision and must complete a self-report of affective commitment and customer orientation while the supervisors provide ratings of PCSP for their 2–6 subordinates. The supervisor and subordinates completed the questionnaires at the same time in different rooms, and none of them could see the content of each other’s questionnaire. They returned the completed questionnaire directly to the principal investigators in order to assure confidential treatment of their individual responses. A total of 85 supervisors and 444 subordinates took part in this survey. Prior to the investigation, the questionnaires were pilot tested for reliability, with Cronbach’s reliability coefficient α scores of four constructs, 0.97, 0.94, 0.91, and 0.90, exceeding the minimum acceptable standard 0.7 as suggested by Nunnally and Bernstein (Nunnally and Bernstein, 1994). The validity of the questionnaire was examined prior to the administration by calculating the data analysis.

By using SPSS21.0, SmartPLS 3, Structure Equation Model (SEM), a multivariate statistical analysis method was conducted with a valid sample of 264 hotel employees and their 62 supervisors.

### Measurement Tools

The hypothesized model measured four latent variables: Abusive Supervision (AS), Affective Commitment (AC), Customer Orientation (CO), and PCSP.

Four existing measurement tools were utilized for the purposes of this study. The criteria for adopting the particular tools are mainly their validity and reliability qualities and their prior utilization in the service industry studies or hospitality and tourism-related studies.

#### Abusive Supervision

The study used the 15-item scale developed by Tepper (Tepper, 2000) to measure subordinates’ perceptions of supervisors’ abusive behaviors, which had been widely used in human resource management studies (Bortolon et al., 2019; Watkins et al., 2019) and had been used to investigate organizational behaviors in the hospitality industry (Park and Kim, 2019).

#### Affective Commitment

This study used the Affective Commitment Scale developed by Allen and Meyer (Allen and Meyer, 1990), which included 8 items and adopted a positive question method. The validity and reliability of this scale had been confirmed by many studies (Qazi et al., 2019), including some studies in hospitality and tourism fields (Günlü et al., 2010).

#### Customer Orientation

The scale measuring customer orientation was adapted from the 5-item scale developed by Thomas (Thomas et al., 2001), which had been used by other researchers with reasonable reliability and validity (Kadic-Maglajlic et al., 2017).

#### Proactive Customer Service Performance

This study used the 7-item scale that was originally developed by Rank et al. (2007), modified by Raub and Liao (2012) to measure employee PCSP. Based on Chinese samples, this scale was also used in the hospitality study with adequate reliability and validity qualities (Huo et al., 2019).

The 35 items, measuring the four latent variables as included in the hypothesized model, are exhibited in Table 1 with the descriptive statistics.

**TABLE 1:** Model items and their descriptive statistics (35 items).

Construct	Item’s label	Question item	s.d.	Mean
Abusive Supervision	AS1	Ridicules me	0.896	1.70
	AS2	Tells me my thoughts or feelings are stupid	0.898	1.73
	AS3	Gives me the silent treatment	0.905	1.72
	AS4	Puts me down in front of others	0.894	1.67
	AS5	Invades my privacy	0.873	1.65
	AS6	Reminds me of my past mistakes and failures	0.984	1.82
	AS7	Doesn’t give me credit for jobs requiring a lot of effort	0.981	1.89
	AS8	Blames me to save himself/herself embarrassment	0.983	1.99
	AS9	Breaks promises he/she makes	0.972	1.83
	AS10	Expresses anger at me when he/she is mad for other reasons	0.997	1.90
	AS11	Makes negative comments about me to others	0.886	1.67
	AS12	Is rude to me	0.838	1.64
	AS13	Does not allow me to interact with coworkers	0.859	1.65
	AS14	Tells me I’m incompetent	0.904	1.74
	AS15	Lies to me	0.862	1.66
Affective Commitment	AC1	I would be very happy to spend the rest of my career with this organization	0.933	3.66
	AC2	I enjoy discussing my organization with others outside it	1.047	3.35
	AC3	I really feel as if this organization’ problems are my own	0.957	3.61
	AC4	I think that I couldn‘t easily as attached to another organization as I am to this organization	0.999	3.58
	AC5	I feel like part of a family at my organization	0.996	3.91
	AC6	I feel emotionally attached to this organization	1.113	3.41
	AC7	This organization has a great deal of personal meaning for me	0.823	3.62
	AC8	I feel a strong sense of belonging to my organization	0.896	3.63
Customer Orientation	CO1	Tries to figure out a customer’s needs	0.765	3.98
	CO2	Has the customer’s best interests in mind	0.870	3.91
	CO3	Takes a problem-solving approach in selling services to customers	0.836	3.97
	CO4	Recommends services that are best suited to solving problems	0.850	3.86
	CO5	Tries to find out which kinds of services would be most helpful to customers	0.849	3.97
PCSP	PCSP1	My staff member anticipates issues or needs customers might have and proactively develops solutions	0.863	3.80
	PCSP2	My staff member proactively shares information with customers to meet their financial needs	0.952	3.64
	PCSP3	My staff member uses own judgment and understanding of risk to determine when to make exceptions or improve solutions	1.007	3.61
	PCSP4	My staff member takes ownership by following through with the customer interaction and ensures a smooth transition to other service representatives	0.872	3.94
	PCSP5	My staff member actively creates partnerships with other service representatives to better service customers	0.792	3.99
	PCSP6	My staff member takes initiative to communicate client requirements to other service areas and collaborates in implementing solutions	0.840	3.97
	PCSP7	My staff member proactively checks with customers to verify that customer expectations have been met or exceeded	0.836	3.96

*1 = strongly disagree; 2 = disagree; 3 = neither disagree nor agree; 4 = agree; 5 = strongly agree.*

## Results

Structural equation modeling (SEM) was used to analyze the data and test the hypothesis (Hair et al., 2019; Hair, 2020). Structural equation modeling makes it possible to study the complex structural relationships among many variables by predicting the dependent variables (Hair et al., 2016; Hallak et al., 2018; Palos-Sanchez et al., 2019). Among various methods of structural equation modeling, partial least square structural equation modeling (PLS-SEM) is used for exploratory research (Reyes-Menendez et al., 2019; Gamil et al., 2020) to test the theoretical framework from the perspective of prediction (Ringle et al., 2020). Using PLS-SEM is generally recommended when the sample size is small and the data are not normally distributed (Reinartz et al., 2009; Richter Nicole et al., 2016; Palos-Sanchez et al., 2019). The theoretical framework used for the test of this study is relatively new, and the sample size is not large, so we decided to use the PLS-SEM method and smartpls 3 software (Ringle et al., 2015) for the data analysis and hypothesis test.

### Sample Characteristics

Of the 444 paired questionnaires distributed, 264 valid questionnaires were collected, thus achieving a response rate of 59.46%. The demographic profile of the respondents (subordinates) according to the variables of gender, age, and educational background are exhibited in Table 2. Of these 264 respondents, 68.18% were female and 64.02% were less than 30 years old, which reflects the real status of employment for the hotel industry in China, that is, most hotel staff in China are young women. As far as the educational background is concerned, 65.91% of respondents had a high school or lower education, which indicates that the educational level of hotel employees is fairly low.

**TABLE 2:** Demographic profile of the respondents (subordinates).

	Frequency	Percentage
**Gender**		
Male	84	31.82
Female	180	68.18
**Age**		
18-25	83	31.44
26-30	86	32.57
31-35	28	10.61
36-40	30	11.36
Over 41	37	14.02
**Educational background**		
High school or less	174	65.91
College degree	76	28.79
Bachelor degree	12	4.54
Master degree	2	0.76

### Measurement Model Evaluation

The reliability of the structures was evaluated with standardized factor loads, composite reliability (CR) values, and Cronbach’s alpha. Table 3 proves that all the standardized factor loadings of each construct except AC2 and PCSP2 are higher than the threshold 0.70 (Hair et al., 2016). The loading values of AC2 and PCSP2 are between 0.672 and 0.679, higher than 0.60, and hence the measurements are retained. The CR values are between 0.921 and 0.969, exceeding the threshold of 0.70 (Hair et al., 2016). Cronbach’s alpha coefficients are between 0.901 and 0.966, which is above the threshold of 0.7. It shows that the analyzed constructs are reliable.

**TABLE 3:** Reliability and Convergent validity of the measurement model.

Construct	Question item	Std. Loadings	CA	CR	AVE
Abusive Supervision	AS1: Ridicules me	0.777	0.966	0.969	0.677
	AS2: Tells me my thoughts or feelings are stupid	0.825			
	AS3: Gives me the silent treatment	0.847			
	AS4: Puts me down in front of others	0.886			
	AS5: Invades my privacy	0.852			
	AS6: Reminds me of my past mistakes and failures	0.830			
	AS7: Doesn’t give me credit for jobs requiring a lot of efforts	0.770			
	AS8: Blames me to save himself/herself embarrassment	0.746			
	AS9: Breaks promises he/she makes	0.858			
	AS10: Expresses anger at me when he/she is mad for other reasons	0.815			
	AS11: Makes negative comments about me to others	0.862			
	AS12: Is rude to me	0.869			
	AS13: Does not allow me to interact with coworkers	0.801			
	AS14: Tells me I‘m incompetent	0.824			
	AS15: Lies to me	0.763			
Affective Commitment	AC1: I would be very happy to spend the rest of my career with this organization	0.804	0.913	0.929	0.623
	AC2: I enjoy discussing my organization with others outside it	0.679			
	AC3: I really feel as if this organization’s problems are my own	0.866			
	AC4: I think that I couldn’t easily as attached to another organization as I am to this organization	0.822			
	AC5: I feel like part of a family at my organization	0.826			
	AC6: I feel emotionally attached to this organization	0.748			
	AC7: This organization has a great deal of personal meaning for me	0.776			
	AC8: I feel a strong sense of belonging to my organization	0.780			
Customer Orientation	CO1: Tries to figure out a customer’s needs	0.862	0.927	0.944	0.773
	CO2: Has the customer’s best interests in mind	0.870			
	CO3: Takes a problem-solving approach in selling services to customers	0.927			
	CO4: Recommends services that are best suited to solving problems	0.886			
	CO5: Tries to find out which kinds of services would be most helpful to customers	0.847			
Proactive Customer Service Performance	PCSP1: My staff member anticipates issues or needs customers might have and proactively develops solutions	0.806	0.901	0.921	0.627
	PCSP2: My staff member proactively shares information with customers to meet their financial needs	0.672			
	PCSP3: My staff member uses own judgment and understanding of risk to determine when to make exceptions or improve solutions	0.737			
	PCSP4: My staff member takes ownership by following through with the customer interaction and ensures a smooth transition to other service representatives	0.823			
	PCSP5: My staff member actively creates partnerships with other service representatives to better service customers	0.818			
	PCSP6: My staff member takes initiative to communicate client requirements to other service areas and collaborates in implementing solutions	0.801			
	PCSP7: My staff member proactively checks with customers to verify that customer expectations have been met or exceeded	0.869			

*Std. loadings = standardized loadings; CA = Cronbach’s alpha; CR = composite reliability; AVE = average variance extracted.*

For construct validity, convergent validity and discriminant validity were checked, respectively. Convergence validity was checked using the average variance extraction (AVE) value. As shown in Table 3, the AVE values of the constructs are all between 0.623 and 0.773, exceeding the threshold value of 0.50 (Hair et al., 2016). The convergent validity of the constructs used in this study is confirmed.

The discriminant validity was checked using the Fornell-Larcker criteria (Fornell and Larcker, 1981) and the HTMT ratio (Henseler et al., 2015). As shown in Table 4, the square feet of the AVE value in each construct exceed the correlation between any two constructs. In addition, the HTMT values are all lower than the conservative threshold of 0.85, and the confidence interval does not contain the value 1 (Hair et al., 2016). As a result, the discriminative validity of the constructs used in this study is confirmed.

**TABLE 4:** Discriminant validity of the measurement model.

Fornell-Larcker criterion					Heterotrait–monotrait ratio (HTMT)			
	AS	AC	CO	PCSP		AS	AC	CO
AS	**0.823**				AS			
AC	−0.387	**0.789**			AC	0.403		
CO	−0.191	0.312	**0.879**		CO	0.197	0.321	
PCSP	−0.188	0.243	0.237	**0.792**	PCSP	0.194	0.260	0.248

AS = Abusive Supervision; AC = Affective Commitment; CO = Customer Orientation; PCSP = Proactive Customer Service Performance; **p < 0.01, ***p < 0.001. The bolded values are the square feet of the AVE value in each construct.

### Structural Model and Hypotheses Testing

We analyzed the collinearity between endogenous structures (VIF), the explanatory power (R^2^) and predictive relevance (Q^2^) of structural models, path coefficients (β), and indirect effects, as shown in Table 5.

**TABLE 5:** Results of hypotheses testing.

	Path coeff(β)	Statistics *t* (β/STDEV)	P Values	BOOTSTRAP 95% CI		Supported
				Lower	Upper	
**H1: AS– > AC**	−0.387	7.964	0.000	−0.472	−0.283	**Yes**
**H2: AC– > CO**	0.312	5.129	0.000	0.176	0.417	**Yes**
**H3: AC– > PCSP**	0.187	2.744	0.006	0.038	0.305	**Yes**
**H4: CO – > PCSP**	0.179	2.699	0.007	0.034	0.296	**Yes**
AS– > AC– > PCSP	−0.072	2.284	0.023	−0.134	−0.014	
AS– > AC– > CO	−0.121	4.229	0.000	−0.176	−0.070	
AS– > AC– > CO– > PCSP	−0.022	2.178	0.030	−0.043	−0.005	
**H5: AC and CO sequentially mediate the relationship between AS and PCSP.**						**Yes**
R^2^: AC = 0.439, CO = 0.607, PCSP = 0.218						
Q^2^: AC = 0.437, CO = 0.605, PCSP = 0.214						

*n = 5000 subsamples. AS = Abusive Supervision; AC = Affective Commitment; CO = Customer Orientation; PCSP = Proactive Customer Service Performance. The bolded terms “H1” to “H5” represent the path between research hypotheses and constructs: H1: AS negatively affects AC; H2: AC positively affects CO; H3: AC positively affects PCSP; H4: CO positively affects PCSP; H5: AC and CO sequentially mediate the relationship between AS and PCSP. The bolded term “Yes” means that the data support this hypothesis.*

First, we detect the multicollinearity between structures. All VIF indexes are lower than 5, which indicates that there is no collinearity problem between the data (Hair et al., 2019).

Secondly, in order to measure the explanatory power of the model, we use the coefficient of determination (R^2^), which represents the variance of one structure’s interpretation of another structure (Hair et al., 2011; Reyes-Menendez et al., 2019). Previous studies considered that R^2^ of 0.67 as substantial, 0.33 as moderate, and 0.19 as weak (Chin, 1998; Gamil et al., 2020). Table 5 shows that the R^2^ values of affective commitment and customer orientation are higher than 0.33, which indicates that their explanatory powers are general. The R^2^ value of PCSP is 0.214, higher than 0.19, indicating that its explanatory power was low but still within the acceptable range.

Thirdly, we use blindfolding to get the cross-validated redundancy index (Q^2^) (Chin, 2010; Dhir et al., 2020). As shown in Table 5, the Q^2^ value of each structure is greater than 0, which indicates that the model has good predictive relevance (Reyes-Menendez et al., 2019).

Finally, the bias-corrected Bootstrap test (Hayes, 2012), with 5,000 bootstrapping subsamples and a confidence interval of 95%, was used to test the significance of the path and obtain the path coefficient to test the hypothesis. As shown in Table 5, P values of all paths are lower than 0.05, 95% of the confidence interval did not contain 0, and so all paths are significant and all the hypotheses proposed are supported by the results.

**FIGURE 2:**
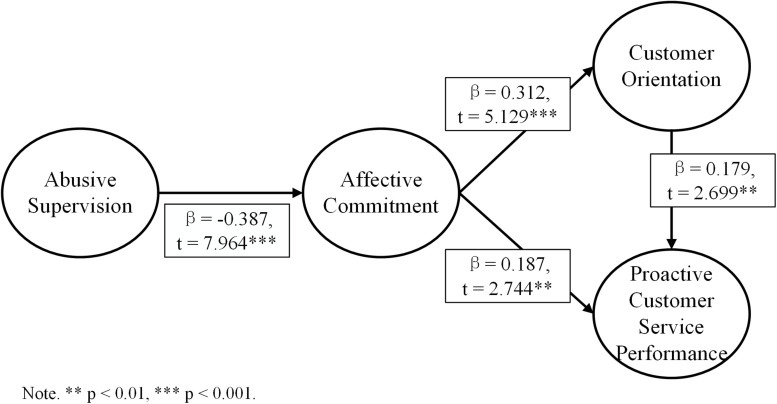
Final research model.

Therefore, all the hypotheses are accepted, and Figure 2 is the final research model.

## Discussion

### Discussions and Implications

Improving employees’ PCSP is essential for the sustainable development of service enterprises, and this has therefore received significant attention from relevant researchers and enterprises. However, there are not many studies that have been made on destructive leadership as one of the important antecedents of PCSP. Therefore, based on the Affective Events Theory and Social Cognitive Theory, this study examined the associations between abusive supervision, affective commitment, customer orientation, and PCSP by using the data collected from hotels in China. With the utilization of structural equation modeling, we gained an in-depth perspective of casual linkages of the above constructs under the circumstances of the tourism and hospitality industry. The empirical research results supported our hypothesized model. The findings of this study have several implications in the field of service science and management practice.

This study has three theoretical implications. Firstly, this study expands the antecedents of PCSP. Previous studies mainly discussed the impact of leadership style and leadership behavior on PCSP from the perspective of positive leadership, while this study explores the influence mechanism and deep motivation of abusive supervision on employees’ PCSP from the perspective of destructive leadership. Consistent with the hypothesis, the findings of this study revealed that abusive supervision has a negative impact on employees’ PCSP. Moreover, affective commitment and customer orientation act as mediators between abusive supervision and PCSP. This supports the judgment drive behavior view of Affective Events Theory that work events affect employees’ work behaviors through their work attitudes such as affective commitments (Weiss and Cropanzano, 1996). As a negative work event, abusive supervision would drive employees to reduce customer orientation by affecting employees’ affective commitments, thereby reducing their proactive customer service performance.

Secondly, previous studies on PCSP are mostly carried out from the perspective of Conservation Of Resources Theory and Social Exchange Theory, while this study provides the perspective of Affective Events Theory. Based on the Affective Events Theory, this study adopts affective commitment as a mediator to study the relationship between abusive supervision and PCSP. Specifically, we found that abusive supervision has a significant negative impact on affective commitment. This finding proves the point that work events affect work attitudes in Affective Events Theory. Past studies have found that there is a negative reciprocal social exchange relationship between abusive supervisors and employees (Cropanzano et al., 2017), and abused employees would take a negative attitude toward the organization as reciprocation for the abusive supervision. Abusive supervision can affect subordinates’ trust of their leaders and reduce their job satisfaction (Tepper, 2007; Velez and Neves, 2017), resulting in a reduction in affective commitment (Tepper, 2007; Wang and Peng, 2015; Ampofo, 2020), which has been confirmed in the context of Chinese hotels in this study. In addition, since abusive supervision is a subjective feeling of subordinates (Tepper, 2000), many supervisors may sometimes not realize their abusive behavior toward their subordinates. However, subordinates who think they are under abusive supervision will reduce their affective commitment toward the organization, which is often overlooked in Chinese service enterprises. Furthermore, we found that affective commitment has a significant positive impact on PCSP. In line with Affective Events Theory, employees’ affective commitment would promote their extra-role and task performance (Loi et al., 2012), prompting them to make behavioral choices other than duty rules that are conducive to organizational development (Paulin et al., 2006; Ben Moussa and El Arbi, 2020), such as PCSP, which is a voluntary service behavior of employees.

The third theoretical implication of this study is to fill the research gap on the relationship between customer orientation and PCSP. This study explores the mediating role of customer orientation in the positive effect of affective commitment on PCSP based on Affective Events Theory and Social Cognitive Theory. We found that affective commitment has a significant positive impact on customer orientation, which supports the view of Affective Events Theory. Affective commitment, as a kind of work attitude, positively promotes employees to make positive work behaviors under judgment-driven behavior. This finding is also in line with those of previous studies that have asserted that employees with higher affective commitment tend to be more customer-oriented (Lombardi et al., 2019; Ribeiro et al., 2020). Customer satisfaction is the key to the sustainable development of service enterprises. For service companies, the degree of customer orientation of employees determines service quality to a certain extent, which is crucial to improving customer satisfaction (Lee et al., 2021). However, customer orientation is a kind of personal behavior of employees, which is uneven. Therefore, increasing employees’ affective commitment to improve customer orientation is beneficial to the long-term development of service enterprises. In addition, we found that customer orientation has a significant positive impact on PCSP. Few studies have involved the relationship between customer orientation and PCSP. This study extends prior study on this issue. Consistent with the self-regulation view of Social Cognitive Theory (Bandura, 1991), employees with high customer orientation have good expectations of service performance (Gazzoli et al., 2012; Ribeiro et al., 2020), and they would therefore self-regulate their service behavior and improve PCSP to obtain better service performance.

Our findings also have significant practical implications in the service industry. Firstly, the results of this study showed that abusive supervision has a negative impact on employees’ PCSP. In fact, the decline of employee service quality and work initiative caused by abusive supervision is widespread in Chinese enterprises. On the one hand, under the influence of the golden mean of Confucianism and high power distance, Chinese employees have seldom protested against their supervisors in person. Chinese employees would rather decrease their PCSP privately to achieve their psychological balance than protest against their supervisors in person, which would be detrimental to the sustainable development of the enterprise (Hofstede, 1980; Yang, 2005). On the other hand, some supervisors cannot realize the negative influence of abusive supervision. They do not know the hurt they had committed, let alone the better communication and management methods. Therefore, it is necessary to know about the tendency of abusive supervise for those candidates according to some investigations in order to exclude those with a high tendency toward abusive supervision. Besides, it is also necessary to establish intervention and monitoring mechanisms to reduce behaviors such as disgrace, ridicule, and cheating.

Secondly, this study found that abusive supervision negatively affects employees’ PCSP by reducing their affective commitment, and employees’ affective commitment positively affects PCSP. In other words, employees with higher affective commitment tend to show higher PCSP. Therefore, in order to improve the PCSP of employees and thus improve organizational performance, measures must be taken to increase the affective commitment of employees. For Chinese service enterprises, it is urgent for the service enterprises to improve the supervisors’ abilities to handle conflict management, team communication, and cooperation in order to improve their level of leadership and enhance employees’ affective commitment.

Thirdly, we found that employees’ affective commitment has a positive impact on PCSP through customer orientation. The higher the employee’s customer orientation, the higher their PCSP. Therefore, it is essential to strengthen the employees’ customer-oriented awareness. Service enterprises should establish a customer-oriented organizational climate and conduct customer-oriented training (Kiffin-Petersen Sandra and Soutar Geoffrey, 2020) for employees to improve their customer orientation.

### Study Limitations and Future Research

This study was based on a longitudinal survey, which can avoid common method variance effectively and make the conclusions more persuasive eventually. However, there are still several limitations to this study. Firstly, just as many current studies, this article explored the status and influence of abusive supervision just from the perspective of employees. Secondly, this study used the measurement tools originally developed by foreign researchers instead of measurement tools developed under Chinese circumstances. Thirdly, this study chose the sample based on the convenience principle that was limited in the employees of hotels and restaurants located in Dezhou and Jinan of Shandong Province. Future studies can explore the relationship between the above constructs under the circumstances of different locations and industries.

## Data Availability Statement

The raw data supporting the conclusions of this article will be made available by the authors, without undue reservation.

## Author Contributions

DZ contributed to the ideas, design, data collection, analyses, literature review, and the writing, reviewing, and editing of the manuscript. CL contributed to the data analyses, literature review, writing of results, and writing of the manuscript. YJ contributed to the ideas, design, data collection, and improvement of the manuscript. All authors contributed to the article and approved the submitted version.

## Conflict of Interest

The authors declare that the research was conducted in the absence of any commercial or financial relationships that could be construed as a potential conflict of interest.
